# Intermittent parathyroid hormone (PTH) promotes cementogenesis and alleviates the catabolic effects of mechanical strain in cementoblasts

**DOI:** 10.1186/s12860-017-0133-0

**Published:** 2017-04-20

**Authors:** Yuyu Li, Zhiai Hu, Chenchen Zhou, Yang Xu, Li Huang, Xin Wang, Shujuan Zou

**Affiliations:** 1 Department of Orthodontics, West China Hospital of Stomatology, Sichuan University, No. 14, 3rd Section, Renmin South Road, Chengdu, 610041 China; 2 State Key Laboratory of Oral Diseases, National Clinical Research Center for Oral Diseases, West China Hospital of Stomatology, Sichuan University, No. 14, 3rd Section, Renmin South Road, Chengdu, 610041 China

**Keywords:** OCCM-30 cell, Intermittent parathyroid hormone (PTH), Mechanical strain, Cementogenesis, Tooth root resorption

## Abstract

**Background:**

External root resorption, commonly starting from cementum, is a severe side effect of orthodontic treatment. In this pathological process and repairing course followed, cementoblasts play a significant role. Previous studies implicated that parathyroid hormone (PTH) could act on committed osteoblast precursors to promote differentiation, and inhibit apoptosis. But little was known about the role of PTH in cementoblasts. The purpose of this study was to investigate the effects of intermittent PTH on cementoblasts and its influence after mechanical strain treatment.

**Results:**

Higher levels of cementogenesis- and differentiation-related biomarkers (bone sialoprotein (BSP), osteocalcin (OCN), Collagen type I (COL1) and Osterix (Osx)) were shown in 1–3 cycles of intermittent PTH treated groups than the control group. Additionally, intermittent PTH increased alkaline phosphatase (ALP) activity and mineralized nodules formation, as measured by ALP staining, quantitative ALP assay, Alizarin red S staining and quantitative calcium assay. The morphology of OCCM-30 cells changed after mechanical strain exertion. Expression of BSP, ALP, OCN, osteopontin (OPN) and Osx was restrained after 18 h mechanical strain. Furthermore, intermittent PTH significantly increased the expression of cementogenesis- and differentiation-related biomarkers in mechanical strain treated OCCM-30 cells.

**Conclusions:**

Taken together, these data suggested that intermittent PTH promoted cementum formation through activating cementogenesis- and differentiation-related biomarkers, and attenuated the catabolic effects of mechanical strain in immortalized cementoblasts OCCM-30.

**Electronic supplementary material:**

The online version of this article (doi:10.1186/s12860-017-0133-0) contains supplementary material, which is available to authorized users.

## Background

External root resorption is a pathological process, which tends to occur following multiple mechanical or chemical stimuli such as infection, trauma or orthodontic treatment and usually begins with the resorption of cementum. This condition may result in pain, swelling and even mobility of the tooth. Treatment alternatives are usually case-dependent, and lack efficacy for the management of external root resorption [1]. Mechanical stimuli with different strain magnitude, frequency, rate or gradients has potent influences on modeling and remodeling of bone and associated cells and signaling pathways [2]. Similar to bone, cementum also undergoes considerable alteration under the influence of mechanical stimuli, and during this procedure, cementoblasts are strain-responsive and have the unique ability to transduce mechanical stimuli into biological events [3]. Moreover, it has been suggested that cementoblasts assist in the process of cementum repair by forming either cellular intrinsic fiber cementum or acellular extrinsic fiber cementum [4].

Endogenous parathyroid hormone (PTH) is the primary regulator of calcium and phosphate metabolism in bone and kidney. Teriparatide/Forteo is a recombinant of human parathyroid hormone [5] that contains the 1–34 amino acid sequence of the complete PTH molecule (PTH (1–34)). The effects of PTH on bone homeostasis are dependent on the mode and dosage of administration. That is, intermittent low-dose PTH increases bone turnover with a greater stimulation of bone formation than bone resorption, whereas continuous high-dose PTH tends to cause catabolic outcomes [6]. Furthermore, the catabolic effects of continuous PTH on cementoblasts have been reported [7, 8]. Evidence has suggested that cementum and bone share many characteristics including gene expression profiles and cell morphologies. However, the effects of intermittent PTH on cementum still remain to be elucidated. We hypothesize that intermittent PTH could also be used to attenuate external root resorption by helping regenerate cementum.

In the present study, we investigated the biological changes of cementoblasts (OCCM-30) after a combined treatment of PTH and mechanical strain by four-point bending system (University of Electronic Science and Technology of China, Chengdu, China). It was shown that intermittent PTH treatment directly regulated cementoblast behavior by detecting the expression of parathyroid hormone receptor type 1 (PTHR1) and some cementogenesis- and differentiation-related biomarkers using qPCR or western blot. Also, intermittent PTH enhanced the ALP activity and the formation of mineralized nodules of OCCM-30 cells. Furthermore, intermittent PTH alleviated the catabolic effect of mechanical strain via regulating the expression of BSP, ALP, OCN, COL1, Runt-related transcription factor 2 (Runx2) and Osx. These results provide the opportunity for evaluating the potential role of intermittent PTH in the cementum regeneration therapies.

## Results

### Intermittent PTH and mechanical strain regulated PTHR1 expression in OCCM-30 cells

**Fig. 1:**
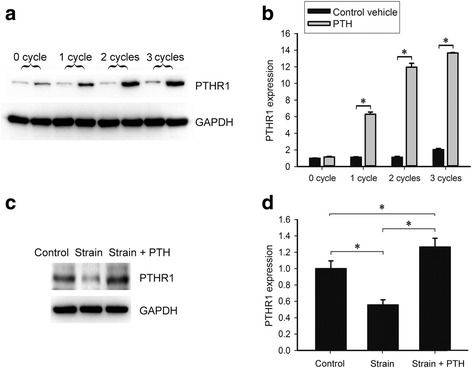
Intermittent PTH treatment and mechanical strain affected the protein level of PTHR1 in OCCM-30 cells. **a** PTH-cycle dependent changes in PTHR1 expression detected by western blot. In each cycle, for the first 6 h, the control group was treated with vehicle culture medium containing acetic acid, and the PTH group received PTH treatment. Then they were both cultured in fresh medium without acetic acid or PTH for 18 h. GAPDH was used as a loading control. **b** Densitometry of bands was performed and normalized to GAPDH. **c** Western blot analysis of PTHR1 and GAPDH levels in OCCM-30 cells in the presence of mechanical strain and intermittent PTH. The Strain + PTH group represented cells that accepted 18 h mechanical strain, followed by 3 cycles of intermittent PTH treatment. The strain group cells were treated with 18 h mechanical strain, followed by 3 cycles of vehicle culture medium. There was neither mechanical strain nor intermittent PTH treatment in the control group. GAPDH was used as an endogenous reference. The experiment was repeated 3 times and the representative result is shown. **d** The bands were quantified and normalized to GAPDH. The control group was set to 1. Bars represent means ± SD of triplicate measurements. * *p* < 0.05

**Fig. 2:**
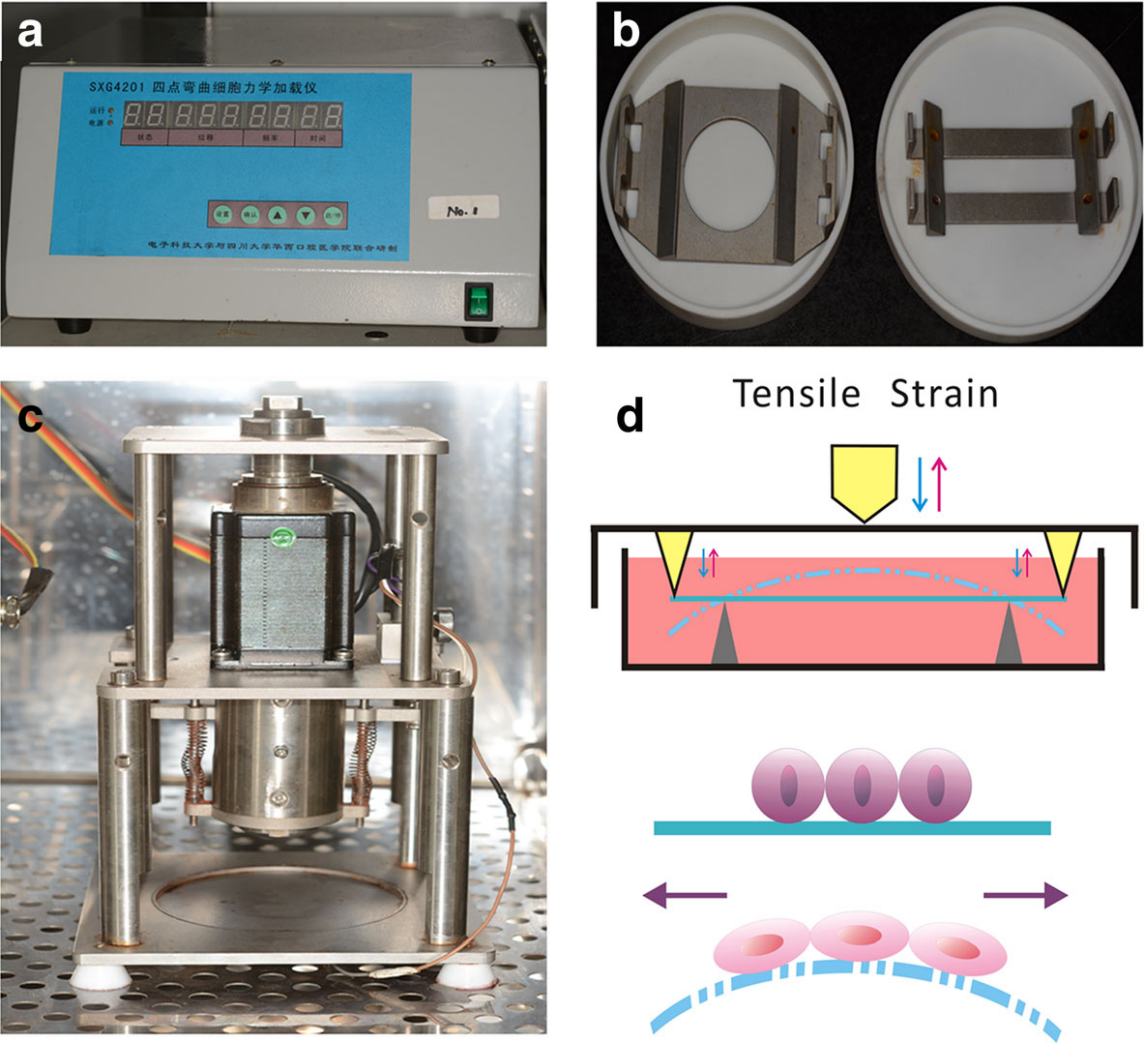
Components and schematic diagram of the uniaxial four-point bending system. The uniaxial four-point bending system is made up of **a** a digital control unit, **b** bending boxes and **c** an actuator. **d** Schematic diagram illustrates how the four-point bending unit works. When the bending box is compressed by the actuator, the cell culture plate is bended by four points to generate tensile strain

It was reported that PTHR1 mRNA was expressed in cememtoblast SV-CM410 subclone [9]. To confirm if OCCM-30 was target cell for PTH, the expression of PTHR1 was examined by western blot (Fig. 1). PTHR1 protein was observed in OCCM-30 cells in the control group, and the upregulation of its expression was PTH-cycle dependent (Fig. 1a and Additional file 1: Figure S1). The increase amounted to 670% after 3 cycles of intermittent PTH, compared to the corresponding control group (Fig. 1b and Additional file 2: Table S1, * *p* < 0.05). To investigate the effects of mechanical strain on OCCM-30, we used the SXG4201 four-point bending device (University of Electronic Science and Technology of China, Chengdu, China; Fig. 2a-c) to apply cyclic mechanical strain to the OCCM-30 cells. OCCM-30 cells were seeded onto the plates and were subjected to 2000 με mechanical strain at 0.5 Hz (loading displacement is 1.12 mm) for 18 h. The deformation of the plates maintained in culture medium caused the attached cells to deform (Fig. 2d). The mechanical strain treatment protocol was all the same throughout the study. Control cultures grew under the same conditions but without the mechanical strain treatment. It was shown that PTHR1 protein expression was decreased by 45% after 18 h mechanical strain (Fig. 1c-d, Additional file 1: Figure S1 and Additional file 2: Table S2, * *p* < 0.05). Additionally, 3 cycles of intermittent PTH treatment after 18 h mechanical strain induced a 26% increase in PTHR1 expression, compared to the control group (Fig. 1c-d, Additional file 1: Figure S1 and Additional file 2: Table S2, * *p* < 0.05). These results suggested that intermittent PTH and mechanical strain had opposite effects on PTHR1 expression and possibly on cementogenic process.

### PTH promoted cementoblast mineralization in OCCM-30 cells

**Fig. 3:**
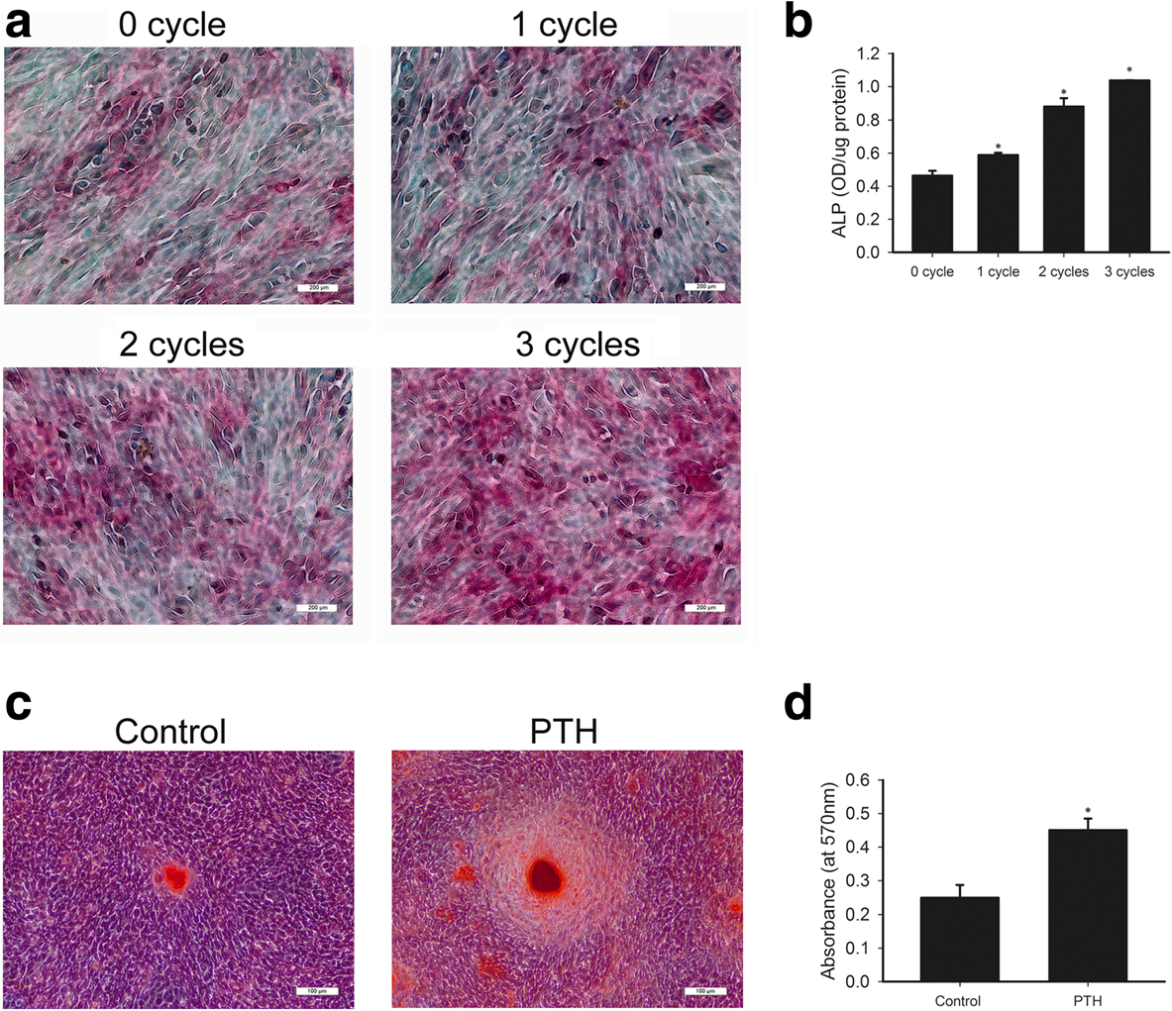
Intermittent PTH administration enhanced ALP activity and the formation of mineralized nodules. **a** Representative images of ALP staining of OCCM-30 cells after treatment with vehicle, 1 cycle, 2 cycles and 3 cycles of intermittent PTH. They together demonstrated a progressively enhanced ALP^+^ staining consistent with the increasing numbers of administration cycles. Scale bar: 200 μm. **b** ALP assay of 0, 1, 2 and 3 cycles of intermittent PTH treated groups. * indicates significant difference between 0 cycle group and other three groups (*p* < 0.05). **c** Microscopic findings of the Alizarin red S staining of the control group and the PTH group. The area of observable mineralized nodules in intermittent PTH treated group was obviously larger in comparison with that of the control group. Scale bar: 100 μm. **d** Alizarin red S extraction assay from the control group and the intermittent PTH treated group. * *p* < 0.05

It was reported that PTH protected against periodontitis-associated bone loss through the regulation of osteoblast activity [10]. We determined mineralization capacity of cementoblasts after intermittent PTH treatment by examining ALP activity and the formation of mineralized nodules. We performed ALP staining, quantitative ALP assay, Alizarin red S staining and quantitative calcium assay. Strong ALP staining was observed after 3 cycles of intermittent PTH treatment (Fig. 3a). As shown in Fig. 3b  and Additional file 2: Table S3, intermittent PTH increased ALP activity in a time-dependent manner in OCCM-30 cells (* *p* < 0.05). Besides, after 10 days of culture, it was shown that 3 cycles of intermittent PTH enhanced the area of mineralized nodules compared to the untreated control (Fig. 3c). For quantitative calcium measurement, stained Alizarin red S was eluted, and the quantification of eluted dye showed enhancement in the intermittent PTH groups, reaching a 1.8-fold increase in the 3 cycles intermittent PTH group as compared to the control group (Fig. 3d and Additional file 2: Table S4, * *p* < 0.05). These results suggested that intermittent PTH could have a promotive role in cementoblast differentiation.

### PTH controlled the expression of cementogenesis-related proteins and regulation of cementoblast differentiation

**Fig. 4:**
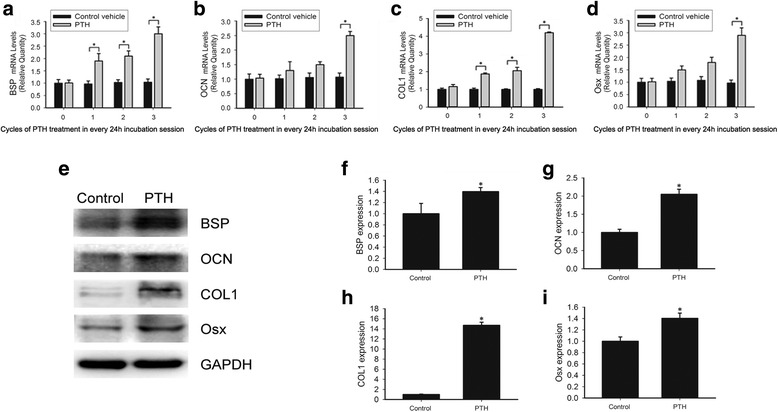
Intermittent PTH markedly increased expression of some cementogenesis-related biomarkers. **a**-**d** The relative mRNA levels of BSP, OCN, COL1 and Osx in vehicle or 0-3 cycles of intermittent PTH treatment groups were determined by qPCR. For the first 6 h in each 24 h cycle, OCCM-30 cells were treated with vehicle or PTH (50 ng/ml) medium, then cells were cultured in fresh medium for the last 18 h in each cycle. Total RNA was subjected to qPCR. Relative mRNA expression levels were normalized to GAPDH as an endogenous reference. **e** The protein levels of BSP, OCN, COL1 and Osx in vehicle or 3 cycles of intermittent PTH treatment groups were determined by western blot. **f**-**i** Densitometry of bands was performed and normalized to GAPDH and fold change was determined relative to the control group. The experiments were repeated 3 times. Bars represent mean ± SD. * *p* < 0.05

Next, we investigated the expression of some mineralization-related proteins involving in cementogenesis and cementoblast differentiation after intermittent PTH treatment. BSP, OCN and COL1 are essential mineralization-related proteins in cementogenesis [11], and regarded as important biomarkers for root regeneration. The mRNA levels of BSP, OCN and COL1 were significantly increased after intermittent PTH treatment. 3 cycles of intermittent PTH induced 2.0-, 1.5- and 4.2-fold increase in BSP, OCN and COL1 expression, respectively (Fig. 4a-c and Additional file 2: Table S5, * *p* < 0.05). Osx is required for cementoblast differentiation and mineralized tissue formation. We observed that intermittent PTH also induced Osx mRNA expression, and the expression of Osx reached maximal levels after 3 cycles of intermittent PTH treatment (Fig. 4d and Additional file 2: Table S5, * *p* < 0.05). Western blot analysis showed that intermittent PTH enhanced the expression of BSP, OCN, COL1 and Osx proteins (Fig. 4e-i, Additional file 3: Figure S2 and Additional file 2: Table S6, * *p* < 0.05). These results indicated that BSP, OCN, COL1 and Osx could play important roles in intermittent PTH-induced cementogenesis.

### Mechanical strain induced morphological changes in OCCM-30 cells

**Fig. 5:**
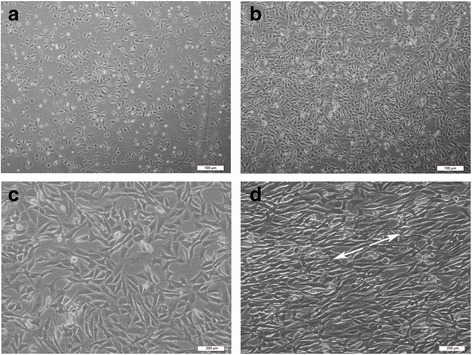
Mechanical strain changed the morphology of OCCM-30 cells. **a**-**d** are representative images of the control group and the mechanical strain-treated group. **a** Four hours after cells being seeded on the plate. **b** Cells that grew for 48 h in the incubator, before the mechanical strain application. Scale bars: 100 μm. **c** The control group that was placed in the bending boxes but did not accept any mechanical strain. **d** The morphological image taken after 18 h mechanical strain. As shown in the representive images, the application of mechanical strain resulted in a tendency of cell realignment corresponding to the direction of the mechanical strain. The arrows decipher the direction of mechanical strain. Scale bars: 200 μm

Mechanical strain is a major regulator of cementum remodeling and resorption [12]. After mechanical strain treatment, the morphological changes of OCCM-30 cells were detected under an optical microscope. Untreated OCCM-30 cells exhibited irregular polygonal- or spindle-shaped morphologies, with the nucleus in the middle of the cytoplasm. And the cells were randomly oriented. However, mechanical strain induced a markedly altered morphology in OCCM-30 cells. Cells under mechanical strain condition were elongated and realigned, conforming to the direction of the mechanical strain (Fig. 5).

### Mechanical strain attenuated cementogenesis- and differentiation-related gene expression

**Fig. 6:**
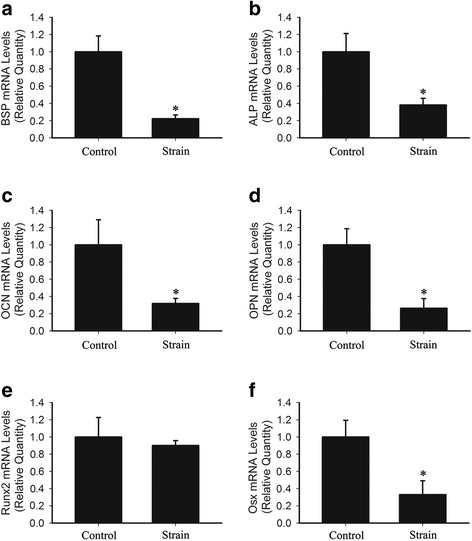
Mechanical strain impaired gene expression of certain cementogenesis-related biomarkers. **a**-**f** The relative mRNA levels of BSP, ALP, OCN, OPN, Runx2 and Osx in OCCM-30 cells in the presence or absence of 18 h mechanical strain. Total RNA was subjected to qPCR. Relative mRNA expression levels were normalized to GAPDH as an endogenous reference. The experiments were repeated 3 times. Bars represent means ± SD. * *p* < 0.05

To further investigate the effects of mechanical strain on cementogenesis in OCCM-30 cells, cememtogenesis-related genes BSP, ALP, OCN and OPN were examined by qPCR. Mechanical strain significantly inhibited cementogenesis-related genes expression in OCCM-30 cells. The expression of BSP decreased by 77% after exposed to mechanical strain of 2000 με for 18 h (Fig. 6a, * *p* < 0.05). Similarly, the expression of ALP, OCN and OPN in mechanical strain group also decreased by 62%, 66% and 74%, respectively (Fig. 6b-d, * *p* < 0.05). We also examined the expression of two transcription factors Runx2 and Osx that are important for cementogenic differentiation. Mechanical strain induced 10% decrease in Runx2 expression (Fig. 6e, *p* > 0.05). However, the expression of Osx decreased by 67% in mechanical strain group (Fig. 6f, * *p* < 0.05). Collectively, these findings suggested that mechanical strain might inhibit cementum formation via attenuating BSP, ALP, OCN, OPN and Osx expression. More detailed data are shown in Additional file 2: Table S7.

### PTH alleviated the inhibition of cementogenesis and cementoblast differentiation by mechanical strain

**Fig. 7:**
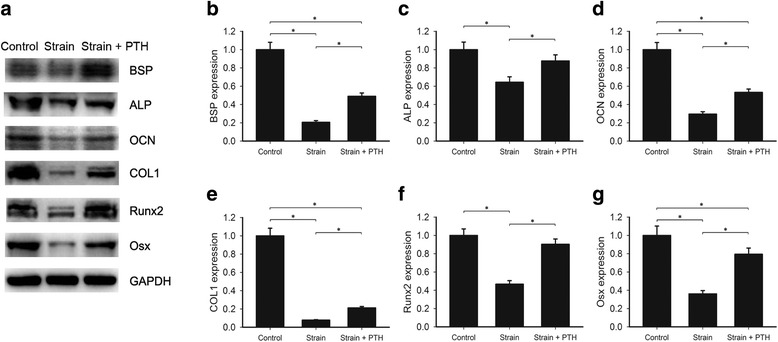
Intermittent PTH attenuated cementogenesis-related gene expression inhibition that was induced by mechanical strain. **a** Western blot analysis of BSP, ALP, OCN, COL1, Runx2 and Osx levels in cells of the control group, the strain group and the strain + PTH group that was treated with mechanical strain and intermittent PTH (also shown in Additional file 4: Figure S3). Except the control group, other two groups were both treated with 18 h mechanical strain, while the control group was placed in the bending box without mechanical strain. Then, the control group and the strain group were exposed to 3 cycles of vehicle treatment. Each cycle comprises 6 h vehicle medium and 18 h fresh medium. Meanwhile, the strain + PTH group were exposed to 3 cycles of intermittent PTH administration. Each cycle comprises 6 h PTH (50 ng/ml) medium and 18 h fresh medium. Results were representative of 3 experiments. **b**-**g** Densitometry of bands was performed and normalized to GAPDH. The control group was set to 1. Bars represent means ± SD of triplicate measurements. * *p* < 0.05

To verify whether or not the impairment of cementogenic ability induced by mechanical strain could be restored by intermittent PTH, OCCM-30 cells were subjected to mechanical strain of 2000 με for 18 h, followed by 3 cycles of intermittent PTH treatment. The expression of BSP, ALP, OCN and COL1 proteins decreased to 20%, 64%, 29% and 8% separately in mechanical strain-only group, which was consistent with our qPCR results that mechanical strain inhibited cementogenesis. However, intermittent PTH significantly upregulated these cementogenesis-related markers, the expression of which rebounded to 49%, 88%, 53% and 21%, respectively, in the strain + PTH group (Fig. 7b-e and Additional file 2: Table S8, * *p* < 0.05). These opposite effects were also observed in differentiation-related proteins. The expression level of Runx2 and Osx in mechanical strain-only group was 47% and 36%, respectively, compared to the control group, while it was 90% and 79% in mechanical strain and PTH group (Fig. 7f-g and Additional file 2: Table S8, * *p* < 0.05). These results demonstrated that intermittent PTH suppressed the adverse effects of mechanical strain on cementum formation through upregulating important biomarkers involved in cementogenic differentiation and cementogenesis processes.

## Discussion

Cementum is a highly responsive mineralized tissue which plays an important role in the development of functional periodontal ligaments. Besides, cementoblastic activities of cementoblasts are thought to be critical in root resorption/repair [4]. Here we investigated the interactive effects of intermittent PTH and mechanical strain on cementogenesis in OCCM-30 cells. Intermittent PTH upregulated PTHR1 expression in OCCM-30 cells. Also, intermittent PTH elevated both mRNA and protein levels of BSP, OCN, COL1 and Osx. However, mechanical strain inhibited cementogenesis as evidenced by the decrease in cementogenesis- and differentiation-related genes expression. The results also demonstrated that intermittent PTH alleviated the mechanical strain-induced inhibition of cementoblast differentiation and cementogenesis based on changes in the expression of cementogenesis- and differentiation-related biomarkers.

PTHR1 [13] is a G protein-coupled receptor (GPCR) that transduces signals into cells via interacting with its two ligands PTH and PTH-related protein (PTHrP). Activation of PTHR1 in osteoblasts and chondrocytes modulated the rates of proliferation and apoptosis, as well as regulated a variety of signaling factors related to the histogenesis and remodeling of bone and cartilage [14]. Our data showed that PTHR1 was endogenously expressed in OCCM-30 cells. And intermittent PTH further upregulated the expression of PTHR1 in OCCM-30 cells. Based on the above, it was concluded that cementoblasts were the target cells of PTH. Along with the result that intermittent PTH activated the cementoblastic activities of cementoblasts, we presume that intermittent PTH can be a new method to promote the regeneration of cementum.

PTH is a major regulator of calcium homeostasis and consequently of bone metabolism. PTH exerts either anabolic or catabolic effects on bone [15, 16] depending upon the dosage and duration of administration. The anabolic effect requires intermittent exposure to low doses of PTH, while continuous exposure to high levels is associated with a catabolic effect [17]. In the present study, we showed that 3 cycles of intermittent PTH treatment enhanced the mineralization capacity of cementoblasts by examining ALP activity and the formation of mineralized nodules. This was further evidenced by quantitative ALP assay and quantitative calcium assay. The cementogenesis- and differentiation-related biomarkers BSP, OCN, COL1 and Osx were also stimulated by intermittent PTH. Previous in vivo studies showed that PTH administration increased cementum width in an osteoporotic rabbit model or improved the formation of newly formed cementum-like tissue in a rat model [18, 19]. Therefore, these findings together suggest that intermittent PTH can be a potential therapeutic agent for root resorption.

Mechanical loading include mechanical strain, compressive strain, fluid shear strain and vibration. Cells perceive and translate mechanical energy into biochemical responses via mechanotransduction pathways [20]. Mechanical energy regulates multiple cellular processes including adhesion, proliferation, differentiation and apoptosis. In order to simulate the action of orthodontic forces that mostly act in a continuous mode accompanying by interruption [21], we selected the four-point bending system to exert cyclic mechanical strain on OCCM-30 cells. Here we showed the regulatory potential of mechanical strain in the biological activities of cementoblasts. First, mechanical strain from the four-point bending device altered the morphology and alignment of cementoblasts. Further, molecular analysis showed that the expression levels of BSP, ALP, OCN, OPN, Runx2 and Osx decreased when OCCM-30 cells were exposed to mechanical strain. Consistently, a previous study showed that mechanical strain of 2000–4000 με inhibited cell proliferation and BSP expression in cementoblasts [22]. A study of human dental pulp stem cells also revealed that mechanical strain suppressed the expression of osteogenesis-related genes bone morphogenetic protein 2 (BMP2), OCN and ALP, as well as odontogenic differentiation-related genes dentin sialophosphoprotein (DSPP), dentin sialoprotein (DSP) and BSP [23]. However, the effects of mechanical strain on cells are controversial. For example, a study demonstrated that after exposure of human intraoral mesenchymal stem and progenitor cells (MSPCs) to cyclic tensile strain, the osteogenic relative factors, such as osteonectin, BMP2, OPN and OCN were upregulated [24]. Considering the complexity of mechanical environment in dental roots under physiologic and pathologic conditions, the elements and mechanisms of mechanical strain that determine the conversion between anabolic and catabolic effects on cementum need further study.

Our study revealed that intermittent PTH increased the expression of BSP, OCN and COL1. However, BSP, ALP, OCN and OPN were inhibited by mechanical strain. BSP, ALP, OCN and OPN are commonly regarded as anabolic markers towards osteoinductivity. BSP is a mineralized, tissue-specific and non-collagenous protein. Its binding to collagen is thought to be important for the initiation of bone mineralization and formation [25]. OCN is a serum marker of osteoblastic bone formation and is believed to act in the bone matrix to regulate mineralization [26]. COL1 is the predominant type of protein that forms the extracellular matrix of bone. It determines the bending and compressive biomechanical properties of cortical bone, and is independent of bone mineral density [27]. Collagens also play vital roles during organ development, wound healing and tissue repair through interacting with growth factors and cytokines. ALP is needed for the normal mineralization during bone modeling and remodeling. It hydrolyzes inorganic pyrophosphate, a natural inhibitor of hydroxyapatite formation, to phosphate. Our research provides a way that PTH can be a potent method to regulate mineral activities in cementoblasts, no matter whether the cells are under the effects of mechanical strain. In our research, the changes of Runx2 and Osx were consistent with the expression of BSP either after intermittent PTH administration or mechanical strain. It was known that Runx2 and Osx were two key transcriptional factors in the differentiation and function of cementoblasts [28–31]. Some studies indicated that Runx2 was an upstream regulator of Osx [32]. Appropriate Runx2 regulatory function required proper association with specific enhancers in the regulatory regions of target genes such as OCN, BSP and OPN, where it recruited other nuclear components to the transcriptional apparatus [33]. Overexpression of Osx greatly accelerated the formation of cellular cementum in a COL1-Osx transgenic mice model [34]. Other studies also demonstrated that Osx regulated cementoblast differentiation through activation of Dkk1 and inhibition of Wnt/β-catenin signaling pathway [28]. It should be emphasized that, in our data, intermittent PTH administration alleviated the catabolic effects induced by mechanical strain. Similarly, a previous study showed that transgenic mice expressing murine Dkk1 had significantly reduced bone mass, while daily PTH administration resulted in comparable increase in bone mass at all skeletal sites [35]. Taken together, we suppose that intermittent PTH has the potential to assist the prevention of tooth root resorption, especially in those cases that improper mechanical loading is a major causative factor.

However, several issues remain to be resolved before the application of intermittent PTH to clinical root resorption treatment. For example, orthodontically induced root resorption is a complex and sterile inflammatory process, including components such as forces, tooth roots, bone, surrounding matrix, cells, and certain biologic messengers. The actions of PTH in this complicated network have not yet been well established. Besides, There are a host of potential contributory factors associated with root resorption among orthodontic patients, including gender, ethnicity, age and genetic disposition of patients, root shape, initial resorption, type of dentition and malocclusion, as well as mechanics and duration of treatment [36]. In light of the complexity of this background, if PTH can be relied on to prevent root resorption regardless of these factors remains to be clarified. Moreover, whether systemic application or local injection of intermittent PTH would be proper for clinical treatment, and what would be the side effects still need further preclinical and clinical data to clarify.

## Conclusions

In summary, this study identified the potential of intermittent PTH to promote cementogenesis. It was shown that intermittent PTH treatment enhanced the mineralization capacity of cementoblasts. Our data also indicated that intermittent PTH improved the expression of cementogenesis- and differentiation-related biomarkers in OCCM-30 cells. Mechanical strain induced visibly morphological changes and suppressed some cementogenesis- and differentiation-related genes expression. Our data also demonstrated that intermittent PTH restrained the inhibition of cementogenesis and cementoblast differentiation by mechanical strain. Taken together, these findings suggest that intermittent PTH can be therapeutically exploited to improve prognosis of tooth root resorption.

## Methods

### Cell culture and reagents

The immortalized mouse cementoblast cell line OCCM-30 was a kind gift from Prof. Somerman and maintained as described previously [37–39]. Briefly, OCCM-30 cells were cultured in Dulbeco’s modified Eagle medium (DMEM) with 10% fetal bovine serum, 100 U/ml penicillin G, and 100 μg/ml streptomycin (Gibco, Grand Island, NY, USA). OCCM-30 cells were incubated in a humidified chamber (5%CO_2_/95% air) at 37 ^0^C. Monolayer cells at 80% confluence were trypsinized and harvested for further study. PTH (1–34) (Bachem, Torrance, CA, USA) was dissolved in 0.1% acetic acid (containing 0.1% bovine serum albumin) according to the manufacturer’s protocol. For intermittent PTH incubation [40], OCCM-30 cells were exposed to 50 ng/ml PTH for the first 6 h in each 24 h treatment session, while the vehicle culture medium of the control group also contained the same concentration of acetic acid but without any PTH. Then cells were all cultured in fresh medium without acetic acid or PTH during the remainder of the session. After 1-3 cycles of treatment, cells were collected and examined.

### Application of mechanical strain

OCCM-30 cells (4 × 10^5^ cells in 1 ml) were seeded onto the loading plates to form a 2 × 2 cm^2^ square. The material of the loading plates was similar to that of cell culture dishes without any surface treatment. Cells were cultured in fresh medium for 48 h to allow cell adhesion, synchronized by 12 h serum starvation and prepared for cyclic uniaxial strain. Mechanical strain was imposed on OCCM-30 cells using the SXG4201 four-point bending device (University of Electronic Science and Technology of China, Chengdu, China; Fig. 2a-c), with the loading plate being maintained in culture medium during loading. The deformation of the plates caused the attached cells to deform (Fig. 2d). Control cultures grew under the same conditions but without the mechanical strain treatment protocol. Cells were subjected to 2000 με mechanical strain at a frequency of 0.5 Hz (As strain is defined as the ratio of the change in length to the original length, 2000 με mechanical strain means 2% changes of the cell length. Correspondingly, the loading displacement of our four-point bending device is 1.12 mm) [22] for 18 h. For the combined treatment of intermittent PTH and mechanical strain, cells were firstly exposed to 18 h mechanical strain in fresh medium, followed by 3 cycles of intermittent PTH treatment.

### Cellular morphological analysis

The morphological changes of OCCM-30 cells after mechanical strain treatment were examined with an Olympus IX70 microscope (Olympus, Tokyo, Japan) at 40× or 100× magnification. 10 fields on each bending plate were randomly selected and images were captured. Cell viability and appearance were analyzed by Image-Pro Plus 6.0 software.

### qPCR

Total RNA was extracted from OCCM-30 cells using Trizol (Invitrogen, Carlsbad, CA, USA). cDNA was synthesized from 1 μg of total RNA as a template, using PrimeScript™ RT reagent Kit with gDNA Eraser (Takara, Tokyo, Japan). qPCR was performed in 20 μL reaction mixtures containing QuantiFast SYBR Green PCR buffer (Qiagen, Valencia, CA, USA), gene-specific primers and cDNAs on a MyIQ thermocycler (Bio-Rad, Hercules, CA, USA). The primer pairs were listed (Table 1). Target gene expression was normalized to GAPDH and analyzed with the use of MyIQ software (Bio-Rad, Hercules, CA, USA).

**Table 1:** Sequences of primers used in qPCR

Gene	Forward primer sequence (5’–3’)	Reverse primer sequence (5’–3’)
GAPDH	GACATCAAGAAGGTGGTGAAGC	GAAGGTGGAAGAGTGGGAGTT
ALP	CCAACTCTTTTGTGCCAGAGA	GGCTACATTGGTGTTGAGCTTTT
BSP	TAGGAGTTTCCAGGTTTCTGATGA	CTGCCCTTTCCGTTGTTGTC
COL1	CTGGCGGTTCAGGTCCAAT	TTCCAGGCAATCCACGAGC
OCN	TGCTTGTGACGAGCTATCAG	GAGGACAGGGAGGATCAAGT
OPN	TAGGAGTTTCCAGGTTTCTGATGA	CTGCCCTTTCCGTTGTTGTC
Osterix	CTCACCAGGTCCAGGCAACA	GGAGCAAAGTCAGATGGGTAAGTAG
Runx2	GGACGAGGCAAGAGTTTCACC	GAGGCGATCAGAGAACAAACTAGG

### Western blot analysis

Cells were rinsed with PBS, trypsinized and collected by centrifugation at 4200 rpm and 4 °C for 5 min. Cell lysis was obtained by incubating in lysis containing RIPA, 1 mM PMSF and complete EDTA-free protease inhibitor cocktail (Roche, Mannheim, Germany). Equivalent amounts of protein (20–40 μg) were subjected to SDS-PAGE (Beyotime, Shanghai, China) and transferred onto a PVDF membrane (Millipore, Bedford, MA, USA). The membrane was blocked with 5% non-fat dried milk in TBST for 1 h and incubated with specific antibodies at 4 °C overnight. Proteins were detected using HRP-conjugated secondary antibodies and visualized using an enhanced chemiluminescence kit (Millipore, Bedford, MA, USA). The intensity of each band was quantified using Quantity One software (Bio-Rad, Hercules, CA, USA) after normalization to GAPDH. For immunoblotting, anti-PTHR1 antibody (Abcam, Cambridge, UK), anti-Runx2 antibody (Abcam, Cambridge, UK), anti-Osx antibody (Abcam, Cambridge, UK), anti-COL1 antibody (Abcam, Cambridge, UK), anti-OPN antibody (Abcam, Cambridge, UK), anti-ALP antibody (Biorbyt, Cambridge, UK), anti-OCN antibody (Biorbyt, Cambridge, UK), and anti-BSP antibody (Biorbyt, Cambridge, UK) were used at 1:1000 dilution.

### ALP staining

ALP activity was performed using an ALP staining kit (Jiancheng, Nanjing, China) according to the manufacturer’s protocol. Briefly, cells were fixed with 10% formalin for 5 min and rinsed in deionized water for 30s. The samples were stained with ALP substrate solution for 15 min at 37 °C in the dark. The stain was removed by washing with deionized water for 30s, and cells were counterstained with hematoxylin. ALP staining intensity was observed under an Olympus IX70 microscope (Olympus, Tokyo, Japan) and analyzed by Image-Pro Plus 6.0 software (Media Cybernetics, Silver Spring, MD, USA).

### Quantitative ALP assay

Corresponding to ALP staining, for quantitative ALP assay, OCCM-30 cells were also treated with 0 cycle, 1 cycle, 2 cycles and 3 cycles of intermittent PTH. The cells were then evaluated for ALP activity. Briefly, cells were rinsed three times with PBS, collected and lysed with ultrasound. The total protein content of these samples was measured by the bicinchoninic acid (BCA) method, using a protein assay kit (Beyotime, Shanghai, China). Then ALP was determined according to a quantitative ALP assay kit (Beyotime, Shanghai, China). The solution mixed by samples and ALP assay working solution was distributed at 100 μL per well on a 96-well plate, and was incubated for 30 min at 37 °C. The optical density (OD) was measured at 405 nm. The ALP activity was normalized to the total protein concentration and was calculated as OD per μg of the proteins.

### Alizarin red S staining and quantitative calcium assay

The Alizarin red S assay was used to determine the degree of mineralization in intermittent PTH and vehicle cultures. The mineralization solution contained ascorbic acid (50 μM), dexamethasone (100 nM), β-glycerophosphate (10 mM) (Sigma, St. Louis, MO, USA) and 10% fetal bovine serum (FBS) in DMEM. 50 ng/ml PTH was dissolved in 0.1% acetic acid in the PTH mineralization solution, while the vehicle mineralization solution contained 0.1% acetic acid without PTH. OCCM-30 cells were seeded in 6-well plates at a density of 1 × 10^5^ cells per well. They were exposed to PTH or vehicle mineralization solution for 3 cycles. Then cells were cultured in mineralization solution without acetic acid or PTH for 7 days. After 10 days of culture, OCCM-30 cells were washed with PBS and fixed with 4% paraformaldehyde at room temperature for 15 min for subsequent Alizarin red S staining (2% (w/v) Alizarin red S (Sigma-Aldrich, St. Louis, MO, USA) solution; pH 4.2), which was conducted (2 ml/each well) for 5 min at room temperature. Following staining, the cells were rinsed with deionized water. The formation of mineralized nodules was observed under an Olympus IX70 microscope (Olympus, Tokyo, Japan) and analyzed by Image-Pro Plus 6.0 software (Media Cybernetics, Silver Spring, MD, USA). For quantitative calcium measurement, 10% cetylpyridinium chloride (J&KCHEMICA, Beijing, China) solution was then added to each well for elution of the dye. After incubation at room temperature for 1 h with shaking, samples of the resulting solution were distributed on a 96-well plate. And absorbance was read at 570 nm.

### Statistical analysis

Data were presented as the mean ± standard deviation (SD). Statistical analyses were performed using ANOVA of factorial design (for factorial designed experiments) or one-way ANOVA (for three or more groups), or independent samples *t* test (between two groups). Values of *p* < 0.05 were defined as statistical significance. All statistical analysis was determined by using SPSS 13.0 (SPSS).

## Additional files

Additional file 1: Figure S1. Bands of Fig. 1 (western blot analysis). (TIF 254 kb)

Additional file 2: Table S1. Densitometry for the bands (western blot analysis) of 0, 1, 2 and 3 cycles of intermittent PTH and the corresponding control groups in Fig. 1a-b. **Table S2.** Densitometry for the bands (western blot analysis) of the control group, the strain group and the strain + PTH group in Fig. 1c-d. **Table S3.** Quantitative analysis of ALP activity. Data indicated the levels of the control group, 1, 2 and 3 cycles of intermittent PTH groups respectively in Fig. 3b. **Table S4.** Data of quantitative calcium assay of the control group and 3 cycles of intermittent PTH group in Fig. 3d. **Table S5.** Data indicating the mRNA levels of BSP, OCN, COL1 and Osx of 0, 1, 2 and 3 cycles of intermittent PTH and the corresponding groups in Fig. 4a-d. **Table S6.** Densitometry for the bands (western blot analysis) of the control group and 3 cycles of intermittent PTH in Fig. 4e-i. **Table S7.** Data indicating the mRNA levels of BSP, ALP, OCN, OPN, Runx2 and Osx of the control group and the strain group after 18 h of mechanical strain treatment in Fig. 6a-f. **Table S8.** Densitometry for the bands (western blot analysis) of the control group, the strain group and the strain + PTH group in Fig. 7a-g. Data were presented as mean ± SD. (DOCX 21 kb)

Additional file 3: Figure S2. Bands of Fig. 4 (western blot analysis). (TIF 1478 kb)

Additional file 4: Figure S3. Bands of Fig. 7 (western blot analysis). (TIF 3040 kb)

## Abbreviations

ALP: Alkaline phosphatase
BMP2: Bone morphogenetic protein 2
BSP: Bone sialoprotein
COL1: Collagen type I
DSP: Dentin sialoprotein
DSPP: Dentin sialophosphoprotein
MSPC: Mesenchymal stem and progenitor cell
OCN: Osteocalcin
OPN: Osteopontin
Osx: Osterix
PTH: Parathyroid hormone
PTHR1: Parathyroid hormone receptor type 1
PTHrP: Parathyroid hormone-related protein
Runx2: Runt-related transcription factor 2
