# Supplementary figures and images for: Intermittent parathyroid hormone (PTH) promotes cementogenesis and alleviates the catabolic effects of mechanical strain in cementoblasts
Source: BMC Cell Biol. 2017 Apr 20;18:19. doi: 10.1186/s12860-017-0133-0 (PMC5397739; doi:10.1186/s12860-017-0133-0)

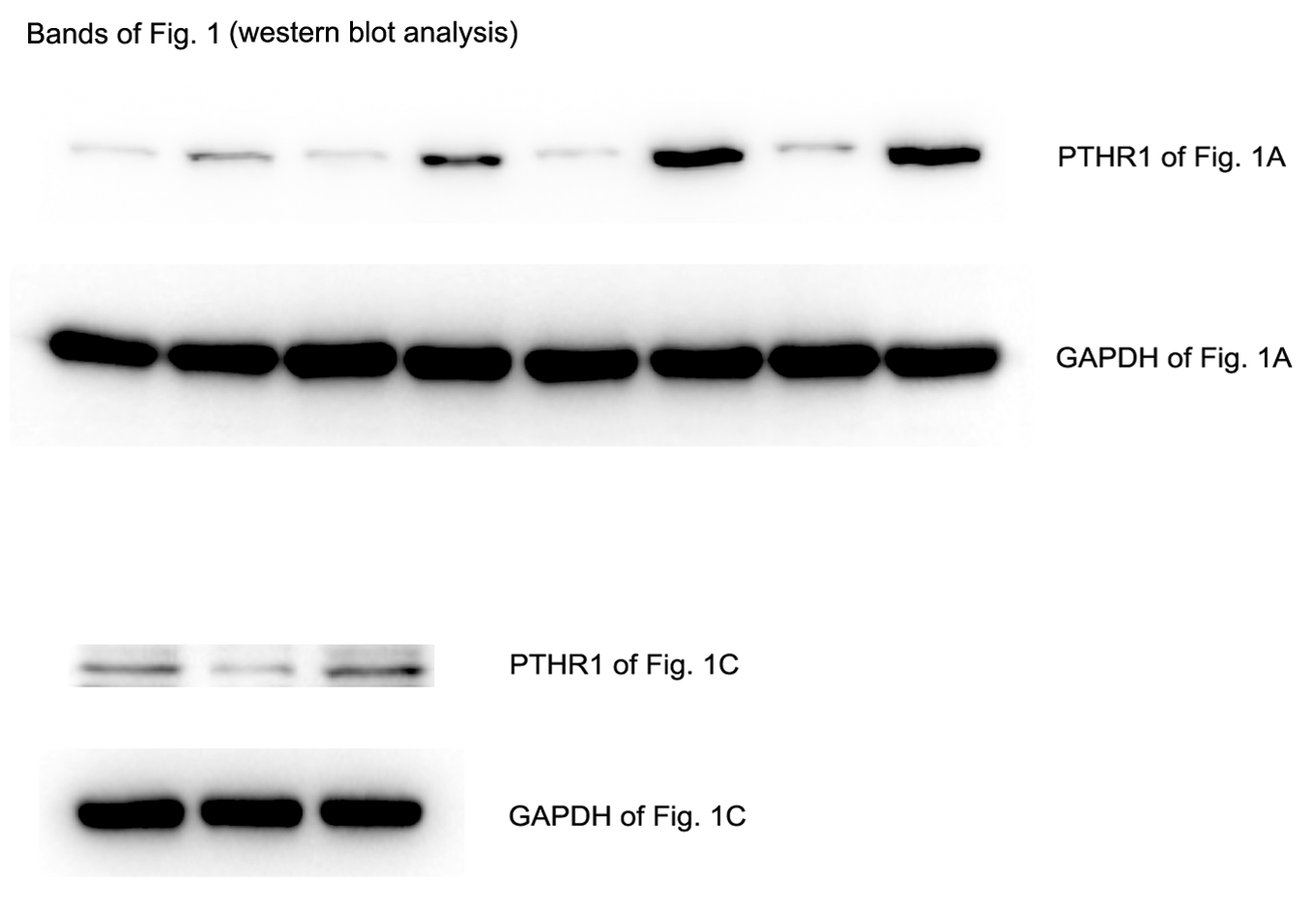

Supplement: Supplementary file 1 — Bands of Fig. 1 (western blot analysis). (TIF 254 kb) [file 12860_2017_133_MOESM1_ESM.tif]

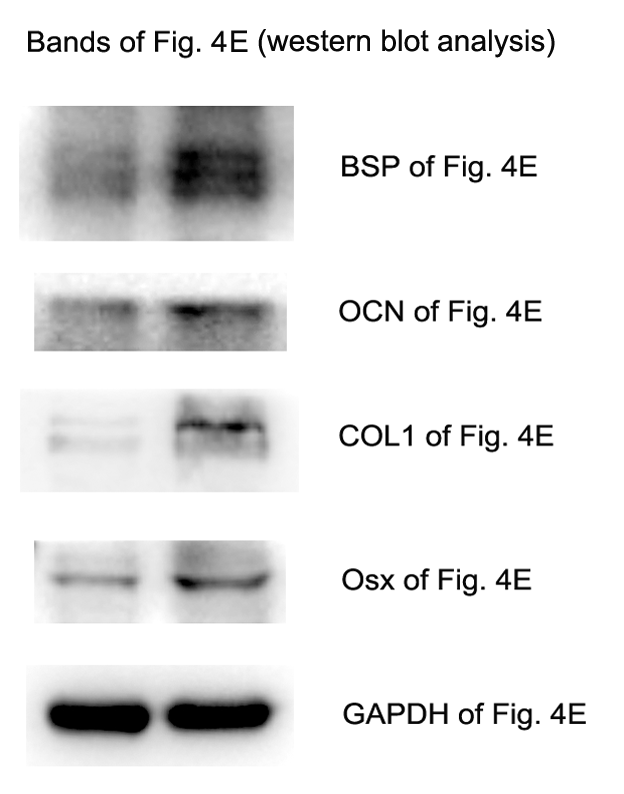

Supplement: Supplementary file 3 — Bands of Fig. 4 (western blot analysis). (TIF 1478 kb) [file 12860_2017_133_MOESM3_ESM.tif]

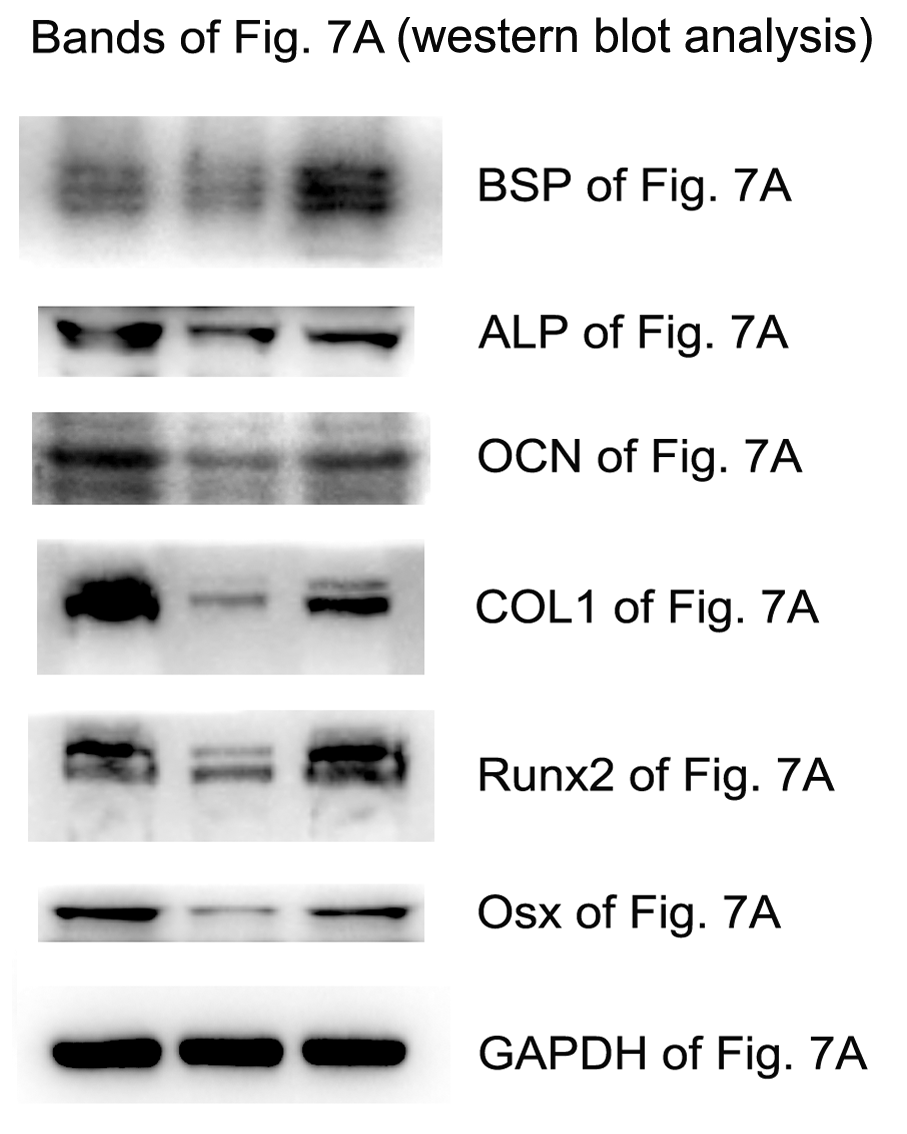

Supplement: Supplementary file 4 — Bands of Fig. 7 (western blot analysis). (TIF 3040 kb) [file 12860_2017_133_MOESM4_ESM.tif]
