# Supplementary material for: Intermittent parathyroid hormone (PTH) promotes cementogenesis and alleviates the catabolic effects of mechanical strain in cementoblasts
Source: BMC Cell Biol. 2017 Apr 20;18:19. doi: 10.1186/s12860-017-0133-0 (PMC5397739; doi:10.1186/s12860-017-0133-0)
Supplement: Supplementary file 2 — Densitometry for the bands (western blot analysis) of 0, 1, 2 and 3 cycles of intermittent PTH and the corresponding control groups in Fig. 1a-b. Table S2. Densitometry for the bands (western blot analysis) of the control group, the strain group and the strain + PTH group in Fig. 1c-d. Table S3. Quantitative analysis of ALP activity. Data indicated the levels of the control group, 1, 2 and 3 cycles of intermittent PTH groups respectively in Fig. 3b. Table S4. Data of quantitative calcium assay of the control group and 3 cycles of intermittent PTH group in Fig. 3d. Table S5. Data indicating the mRNA levels of BSP, OCN, COL1 and Osx of 0, 1, 2 and 3 cycles of intermittent PTH and the corresponding groups in Fig. 4a-d. Table S6. Densitometry for the bands (western blot analysis) of the control group and 3 cycles of intermittent PTH in Fig. 4e-i. Table S7. Data indicating the mRNA levels of BSP, ALP, OCN, OPN, Runx2 and Osx of the control group and the strain group after 18 h of mechanical strain treatment in Fig. 6a-f. Table S8. Densitometry for the bands (western blot analysis) of the control group, the strain group and the strain + PTH group in Fig. 7a-g. Data were presented as mean ± SD. (DOCX 21 kb) [file 12860_2017_133_MOESM2_ESM.docx]

**Table S1.** Densitometry for the bands (western blot analysis) of 0, 1, 2 and 3 cycles of intermittent PTH and the corresponding control groups in Fig. 1A-B. **Table S2.** Densitometry for the bands (western blot analysis) of the control group, the strain group and the strain + PTH group in Fig. 1C-D. **Table S3.** Quantitative analysis of ALP activity. Data indicated the levels of the control group, 1, 2 and 3 cycles of intermittent PTH groups respectively in Fig. 3B. **Table S4.** Data of quantitative calcium assay of the control group and 3 cycles of intermittent PTH group in Fig. 3D. **Table S5.** Data indicating the mRNA levels of BSP, OCN, COL1 and Osx of 0, 1, 2 and 3 cycles of intermittent PTH and the corresponding groups in Fig. 4A-D. **Table S6.** Densitometry for the bands (western blot analysis) of the control group and 3 cycles of intermittent PTH in Fig. 4E-I. **Table S7.** Data indicating the mRNA levels of BSP, ALP, OCN, OPN, Runx2 and Osx of the control group and the strain group after 18h of mechanical strain treatment in Fig. 6A-F. **Table S8.** Densitometry for the bands (western blot analysis) of the control group, the strain group and the strain + PTH group in Fig. 7A-G. Data were presented as mean ± SD.

Table S1 (Data of densitometry in Fig. 1A-B)

| Groups | 0 cycle control | 0 cycle  PTH | 1 cycle  control | 1 cycle  PTH | 2 cycles  control | 2 cycles  PTH | 3 cycles  control | 3 cycles  PTH |
| --- | --- | --- | --- | --- | --- | --- | --- | --- |
| Mean ± SD | 0.04±0.001 | 0.05±0.004 | 0.05±0.003 | 0.27±0.01 | 0.05±0.005 | 0.52±0.02 | 0.09±0.006 | 0.60±0.002 |

Table S2 (Data of densitometry in Fig. 1C-D)

| Groups | Control | Strain | Strain + PTH |
| --- | --- | --- | --- |
| Mean ± SD | 0.11±0.01 | 0.06±0.01 | 0.14±0.01 |

Table S3 (Data of optical density in Fig. 3B)

| Groups | 0 cycle  control | 1 cycle  PTH | 2 cycles  PTH | 3 cycles  PTH |
| --- | --- | --- | --- | --- |
| Mean ± SD | 0.46±0.03 | 0.59±0.01 | 0.88±0.05 | 1.04±0.001 |

Table S4 (Data of optical density in Fig. 3D)

| Groups | Control | PTH |
| --- | --- | --- |
| Mean ± SD | 0.25±0.04 | 0.45±0.03 |

Table S5 (Data of qPCR in Fig. 4A-D)

| Groups  Mean±SD  Biomarkers | 0 cycle control | 0 cycle  PTH | 1 cycle  control | 1 cycle  PTH | 2 cycles  control | 2 cycles  PTH | 3 cycles  control | 3 cycles  PTH |
| --- | --- | --- | --- | --- | --- | --- | --- | --- |
| BSP | 1.01±0.25 | 1.22±0.19 | 0.93±0.23 | 1.94±0.25 | 1.33±0.13 | 2.1±0.19 | 1.54±0.26 | 3.44±0.28 |
| OCN | 1.12±0.23 | 1.24±0.30 | 1.51±0.14 | 1.34±0.26 | 1.31±0.26 | 1.54±0.10 | 1.63±0.21 | 2.54±0.13 |
| COL1 | 1.00±0.07 | 1.16±0.12 | 1.00±0.04 | 2.05±0.19 | 1.04±0.07 | 1.87±0.06 | 1.03±0.04 | 4.20±0.04 |
| Osx | 1.23±0.22 | 1.33±0.23 | 1.32±0.13 | 1.55±0.16 | 1.41±0.26 | 1.85±0.20 | 0.91±0.25 | 2.94±0.30 |

Table S6 (Data of densitometry in Fig. 4E-I)

| Groups  Mean±SD  Biomarkers | Control | PTH |
| --- | --- | --- |
| BSP | 0.51±0.09 | 0.71±0.04 |
| OCN | 0.20±0.02 | 0.41±0.03 |
| COL1 | 0.05±0.004 | 0.76±0.03 |
| Osx | 0.12±0.01 | 0.17±0.01 |

Table S7 (Data of qPCR in Fig. 6A-F)

| Groups  Mean±SD  Biomarkers | Control | Strain |
| --- | --- | --- |
| BSP | 1.01±0.19 | 0.22±0.04 |
| ALP | 0.99±0.21 | 0.38±0.08 |
| OCN | 0.99±0.29 | 0.32±0.06 |
| OPN | 0.95±0.18 | 0.25±0.11 |
| Runx2 | 1.02±0.25 | 0.92±0.06 |
| Osx | 1.02±0.20 | 0.33±0.16 |

Table S8 (Data of densitometry in Fig. 7A-G)

| Groups  Mean±SD  Biomarkers | Control | Strain | Strain + PTH |
| --- | --- | --- | --- |
| BSP | 0.33±0.03 | 0.07±0.01 | 0.16±0.01 |
| ALP | 0.38±0.03 | 0.24±0.02 | 0.33±0.03 |
| OCN | 0.25±0.02 | 0.07±0.003 | 0.14±0.01 |
| COL1 | 1.61±0.13 | 0.13±0.01 | 0.34±0.02 |
| Runx2 | 0.38±0.03 | 0.12±0.01 | 0.35±0.01 |
| Osx | 0.38±0.02 | 0.14±0.01 | 0.30±0.01 |
